# Advancing Diabetic Retinopathy Screening: A Systematic Review of Artificial Intelligence and Optical Coherence Tomography Angiography Innovations

**DOI:** 10.3390/diagnostics15060737

**Published:** 2025-03-15

**Authors:** Alireza Hayati, Mohammad Reza Abdol Homayuni, Reza Sadeghi, Hassan Asadigandomani, Mohammad Dashtkoohi, Sajad Eslami, Mohammad Soleimani

**Affiliations:** 1 Students’ Research Committee (SRC), Qazvin University of Medical Sciences, Qazvin 34197-59811, Iran; alirezahayati17@yahoo.com; 2 Eye Research Center, Farabi Eye Hospital, Tehran University of Medical Sciences, Tehran 13399-73111, Iran; rezahomayuni1@gmail.com (M.R.A.H.); rsadeghi73@gmail.com (R.S.); hasanasadigandomani0800@gmail.com (H.A.); 3 School of Medicine, Tehran University of Medical Sciences, Tehran 13399-73111, Iran; 4 Students Scientific Research Center (SSRC), Tehran University of Medical Sciences, Tehran 13399-73111, Iran; md1999c@gmail.com; 5 School of Business, Stevens Institute of Technology, Hoboken, NJ 07030, USA; sajad.eslami@gmail.com; 6 Department of Ophthalmology, University of North Carolina at Chapel Hill, Chapel Hill, NC 27599, USA; 7 AI.Health4All Center for Health Equity using ML/AI, College of Medicine, University of Illinois at Chicago, Chicago, IL 60607, USA

**Keywords:** diabetic retinopathy, optical coherence tomography angiography, artificial intelligence, deep learning, machine learning, ophthalmology, screening

## Abstract

**Background/Objectives**: Diabetic retinopathy (DR) remains a leading cause of preventable blindness, with its global prevalence projected to rise sharply as diabetes incidence increases. Early detection and timely management are critical to reducing DR-related vision loss. Optical Coherence Tomography Angiography (OCTA) now enables non-invasive, layer-specific visualization of the retinal vasculature, facilitating more precise identification of early microvascular changes. Concurrently, advancements in artificial intelligence (AI), particularly deep learning (DL) architectures such as convolutional neural networks (CNNs), attention-based models, and Vision Transformers (ViTs), have revolutionized image analysis. These AI-driven tools substantially enhance the sensitivity, specificity, and interpretability of DR screening. **Methods**: A systematic review of PubMed, Scopus, WOS, and Embase databases, including quality assessment of published studies, investigating the result of different AI algorithms with OCTA parameters in DR patients was conducted. The variables of interest comprised training databases, type of image, imaging modality, number of images, outcomes, algorithm/model used, and performance metrics. **Results**: A total of 32 studies were included in this systematic review. In comparison to conventional ML techniques, our results indicated that DL algorithms significantly improve the accuracy, sensitivity, and specificity of DR screening. Multi-branch CNNs, ensemble architectures, and ViTs were among the sophisticated models with remarkable performance metrics. Several studies reported that accuracy and area under the curve (AUC) values were higher than 99%. **Conclusions**: This systematic review underscores the transformative potential of integrating advanced DL and machine learning (ML) algorithms with OCTA imaging for DR screening. By synthesizing evidence from 32 studies, we highlight the unique capabilities of AI-OCTA systems in improving diagnostic accuracy, enabling early detection, and streamlining clinical workflows. These advancements promise to enhance patient management by facilitating timely interventions and reducing the burden of DR-related vision loss. Furthermore, this review provides critical recommendations for clinical practice, emphasizing the need for robust validation, ethical considerations, and equitable implementation to ensure the widespread adoption of AI-OCTA technologies. Future research should focus on multicenter studies, multimodal integration, and real-world validation to maximize the clinical impact of these innovative tools.

## 1. Introduction

Diabetic retinopathy (DR) is one of the most prevalent and debilitating complications of diabetes mellitus, representing a leading cause of preventable blindness worldwide. As of recent estimates, approximately 285 million individuals globally are affected by DR, a figure projected to escalate to 600 million by 2040 [1]. This surge is closely tied to the rising incidence of diabetes, underscoring the urgent need for effective screening and management strategies. Early detection and timely intervention are critical, as more than 95% of DR-related vision loss can be prevented or significantly delayed with appropriate treatment [1]. Consequently, regular DR screening is strongly recommended for all patients diagnosed with diabetes to mitigate the risk of severe ocular complications [2]. These vascular alterations disrupt the delicate balance of retinal oxygenation and nutrient supply, leading to progressive retinal damage and vision impairment [3].

Advancements in retinal imaging technology have revolutionized the field of ophthalmology, enhancing the precision and efficacy of early disease diagnosis. Optical Coherence Tomography Angiography (OCTA) has emerged as a transformative tool since its introduction, offering unparalleled non-invasive visualization of the retinal and choroidal vasculature. Unlike traditional imaging modalities such as fluorescein angiography (FA) and fundus photography, OCTA provides three-dimensional, layer-specific retina images without needing contrast agents or pupil dilation [4]. This capability allows for detailed differentiation between the superficial and deep capillary plexus layers, facilitating the detection of subtle microvascular changes associated with DR progression. While current DR screening methods, such as fundus photography and FA, require manual interpretation, which can be time-consuming and subjective, artificial intelligence (AI)-assisted OCTA offers a more efficient and objective approach. AI algorithms can analyze OCTA images rapidly and consistently, potentially increasing screening capacity and reducing diagnostic delays.

The superior imaging capabilities of OCTA have enhanced clinical diagnostics and paved the way for integrating increasingly advanced computational methods. In earlier approaches, traditional machine learning (ML) models relied on handcrafted features and often demonstrated inconsistent performance due to their limited capacity for nuanced image interpretation. By contrast, deep learning (DL) architectures, particularly convolutional neural networks (CNNs), have transformed this landscape through their ability to automatically extract intricate, hierarchically organized features from OCTA data, markedly improving diagnostic accuracy and robustness. More recent CNN-based models, including attention-driven frameworks and ensemble methods, further refine these capabilities by highlighting critical vascular patterns and strengthening model reliability [4]. Beyond CNNs, the emergence of Vision Transformers (ViTs) and related transform ViTs based architectures has introduced an attention-centric paradigm that does not depend solely on local convolutional filters [5]. These transformer models can contextualize the entire image globally, improving interpretability and offering even higher classification precision [6]. Collectively, the evolution from traditional ML to advanced CNNs, attention-based networks, and ViTs has fundamentally elevated the accuracy, sensitivity, and specificity of DR detection, advancing the field toward more reliable and insightful AI-driven solutions [7,8,9,10].

Despite the significant progress, some studies have raised questions about the sensitivity of AI in detecting early-stage DR or specific subtle features compared to expert clinicians [11]. These conflicting findings highlight the need for further research to optimize AI algorithms and validate their performance across diverse clinical settings and patient populations. Automated systems leveraging DL models can process vast amounts of imaging data efficiently, reducing the burden on healthcare professionals and enabling scalable screening programs. Moreover, the non-invasive nature of OCTA combined with AI analytics enhances patient comfort and compliance, addressing key barriers to regular screening [12].

Despite these promising developments, several challenges remain. Variability in OCTA image acquisition protocols, differences in device specifications, and the “black box” nature of many DL models pose significant hurdles for clinical implementation. The “black box” issue refers to the lack of transparency in how DL models make decisions, which can hinder clinician trust and impede widespread adoption. As these models become more complex, understanding the rationale behind their predictions becomes increasingly difficult, making it challenging for healthcare professionals to verify or interpret the results. To address this, explainable AI (XAI) approaches are being developed to provide visualizations, attention maps, or justifications for model decisions, enabling clinicians to evaluate and trust AI outputs [13]. For example, attention mechanisms in ViTs can highlight regions of interest in OCTA images, offering insights into how the model arrived at its diagnosis. This transparency is critical for fostering clinician confidence and ensuring safe, ethical integration of AI into clinical workflows. Additionally, ensuring the generalizability of AI models across diverse populations and clinical settings requires extensive validation through multicenter studies and large-scale datasets [14,15].

This systematic review aims to comprehensively evaluate the current landscape of AI integration with OCTA imaging for the detection and classification of DR. In contrast to other systematic reviews that may focus on broader imaging techniques or AI applications in DR screening, this review concentrates explicitly on the burgeoning field of AI-enhanced OCTA. By analyzing recent advancements, performance metrics, and clinical implications, this review seeks to elucidate the potential and limitations of AI-enhanced OCTA in revolutionizing DR screening and management. Specifically, this review will delve into the application of advanced DL methods, including CNNs and ViTs, to OCTA image analysis and assess their ability to detect subtle microvascular changes characteristic of DR. Furthermore, it will focus on the practical and clinical implications of AI-OCTA systems, evaluating their potential to improve screening workflows and patient outcomes. Understanding these dynamics is crucial for guiding future research, optimizing clinical workflows, and ultimately improving patient outcomes in the global fight against DR.

## 2. Materials and Methods

The current systematic review was conducted according to evidence-based criteria provided by the Preferred Reporting Items for Systematic Reviews and Meta-Analyses (PRISMA) guideline.

### 2.1. Search Strategy

PubMed, Scopus, Embase, and Web of Science were systematically searched to identify relevant articles from the earliest published record until November 2024. Each database was searched using appropriate terms related to OCTA, DR, and AI (Table 1). There was no filter regarding the publication location. We also carried out a manual search by reviewing the references of the included research to reduce the possibility of overlooking any relevant studies.

### 2.2. Eligibility Criteria

Published studies that used AI algorithms to assess OCTA images measuring the retinal and choroidal microvasculature in patients with DR were included in this review if they met the following requirements: (a) written in English; (b) original, peer-reviewed research; and (c) presence of a control group. Studies with the following characteristics were not included: (a) case reports, reviews, book chapters, letters, or conference abstracts; (b) not in English; (c) not original; (d) not human; (e) not having a control group.

### 2.3. Quality Assesment

The quality of the included studies was assessed using the Newcastle-Ottawa Scale (NOS), which evaluates three key domains: selection of study groups, comparability, and ascertainment of exposure. Each study was assigned a score based on predefined criteria, with higher scores indicating better methodological quality. Studies were assessed for adequacy of case definition, representativeness, selection and definition of controls, comparability based on age and other factors, as well as ascertainment of exposure, consistency of measurement, and non-response rate. We included studies that scored 5 or more, with the highest possible score being 9 (Supplement S1).

### 2.4. Data Extraction

Following a thorough primary review of the unique publications that were found, two independent authors (MA and AH) extracted the following data from the included research: (1) first author and publication year; (2) number of images; (3) training database; (4) type of image and modality; (5) outcomes; (6) algorithm/model used; (7) and performance metrics. Any conflicts that arose throughout the data extraction procedure were resolved by the third author (RS).

### 2.5. Comparing AI Models

We evaluated and contrasted the effectiveness of various AI models in identifying DR using OCTA images utilizing the criteria of accuracy, area under the curve (AUC), sensitivity, and specificity. These criteria were chosen for their comprehensive ability to assess the diagnostic efficacy of AI systems. Accuracy measures the proportion of correct results to the total cases examined, thoroughly evaluating the model’s overall precision. The AUC of the Receiver Operating Characteristic (ROC) curve indicates the model’s ability to distinguish across several classes, with higher AUC values denoting enhanced discriminative performance [16]. Sensitivity, or recall, assesses the model’s ability to correctly identify actual positive instances, which is crucial in clinical settings for the timely detection and management of DR [17]. Specificity evaluates the model’s ability to correctly identify true negative cases, reducing the likelihood of false positives and limiting unnecessary interventions [18]. The therapeutic significance of these metrics lies in their ability to balance the trade-offs between detecting disease and preventing overdiagnosis, hence improving patient outcomes through accurate and reliable screening methods [16,17,18].

### 2.6. Dataset Imbalance and Its Impact on Model Bias

The variability in dataset sizes and the disproportionate representation of DR severity levels in the studies we assessed may introduce biases in the training of the AI model. Restricted datasets, as illustrated by Bidwai et al. [19] (*n* = 76) and Khalili Pour et al. [20] (*n* = 78), may lead to overfitting and limit the model’s generalizability. Larger datasets such as ROAD [20] (*n* = 2640) provide a more diverse training set, enhancing model robustness; however, they may still encounter class imbalance. The uneven representation of DR severity levels in these datasets is an obstacle, as models predominantly trained on particular stages may not demonstrate consistent performance across all severity levels. This discrepancy may result in distorted predictions, particularly underrepresenting early-stage DR or disproportionately emphasizing severe cases. Addressing these inequalities through techniques like synthetic oversampling or stratified sampling is crucial for developing unbiased and generalizable AI models for DR detection.

### 2.7. Data and Code Accessibility

This study is a systematic review and does not involve original data collection or AI model development. To ensure reproducibility, we have provided a detailed description of our systematic search strategy, including search terms, databases (PubMed, Scopus, WOS, and Embase), inclusion/exclusion criteria, and data extraction methods. The extracted dataset, including key study characteristics and performance metrics, is available in Table 2 of the manuscript. Additionally, the systematic review protocol has been registered and can be accessed at https://osf.io/q6dt5/ (accessed on 4 February 2025).

## 3. Results

### 3.1. Literature Search

**Figure 1:**
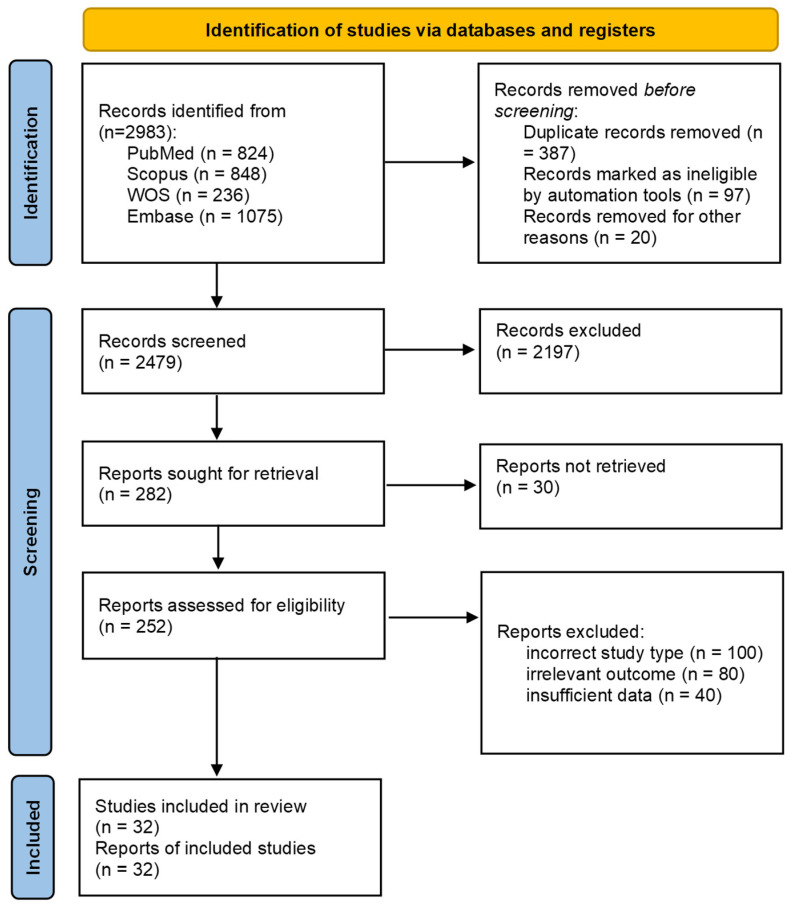
PRISMA flow diagram of the literature review process.

A total of 2983 records were identified across four databases. After removing 387 duplicates and 117 records deemed ineligible by automation or other reasons, 2479 unique articles were screened. Title abstract screening yielded 2197 irrelevant articles. The remaining 282 articles were sought for retrieval. However, 30 articles were not retrieved due to the absence of full text or English full text. Full-text screening was undertaken for the remaining 252 articles. During the full-text screening process, 220 studies were excluded, and finally, 32 articles were included (Figure 1).

### 3.2. Sample Size

Ryu et al. had the most significant sample, with 1118 participants [36]. Following them, Li et al. [38] and Nagasawa et al. [28] used 611 and 491 samples, respectively, employing DenseNet121, EfficientNet b3, and deep convolutional neural network (DCNN) algorithms. Conversely, Bidwai et al. [45] and Khalili Pour et al. [20] used the smallest sample sizes of 76 and 78 for Light GBM and SVM optimized by genetic algorithms, respectively.

### 3.3. Training Databases

**Figure 2:**
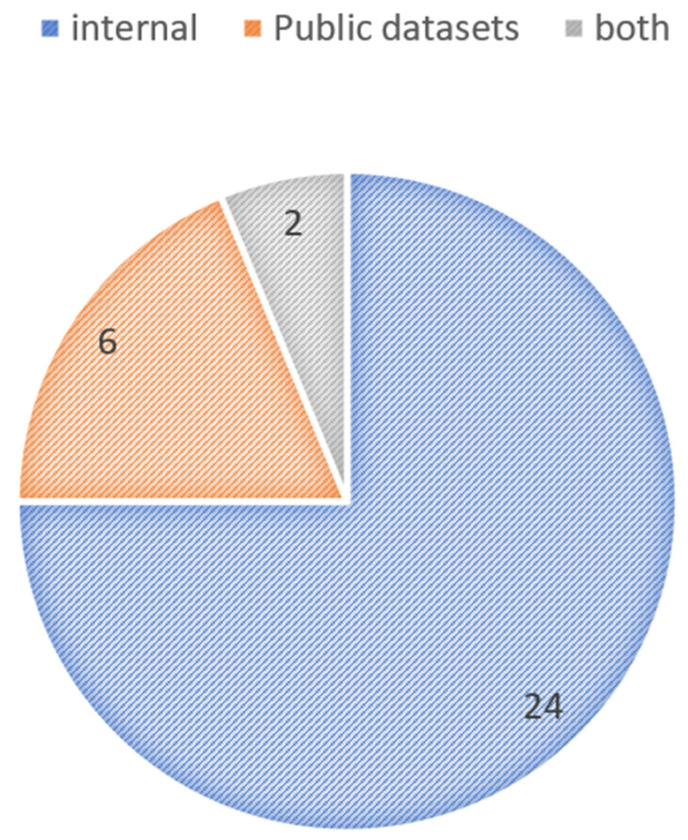
Distribution of studies based on datasets: internal, public, and both.

Based on training databases and recruited patients, we classified the included studies into two groups: (1) internal databases in which patients of an eye center or hospital were recruited, and there was no public access to these databases; (2) studies using the records of online and public databases. Six papers used public datasets such as ROAD, EviRed, and Diabetic Retinopathy and Cataract dataset (DRAC) to conduct their study. A total of 24 studies were conducted based on internal databases, while two studies used both internal and Messidor datasets as their source (Figure 2). Further explanations of the public databases used are presented in Supplement S2.

### 3.4. Algorithm/Model Used

**Figure 3:**
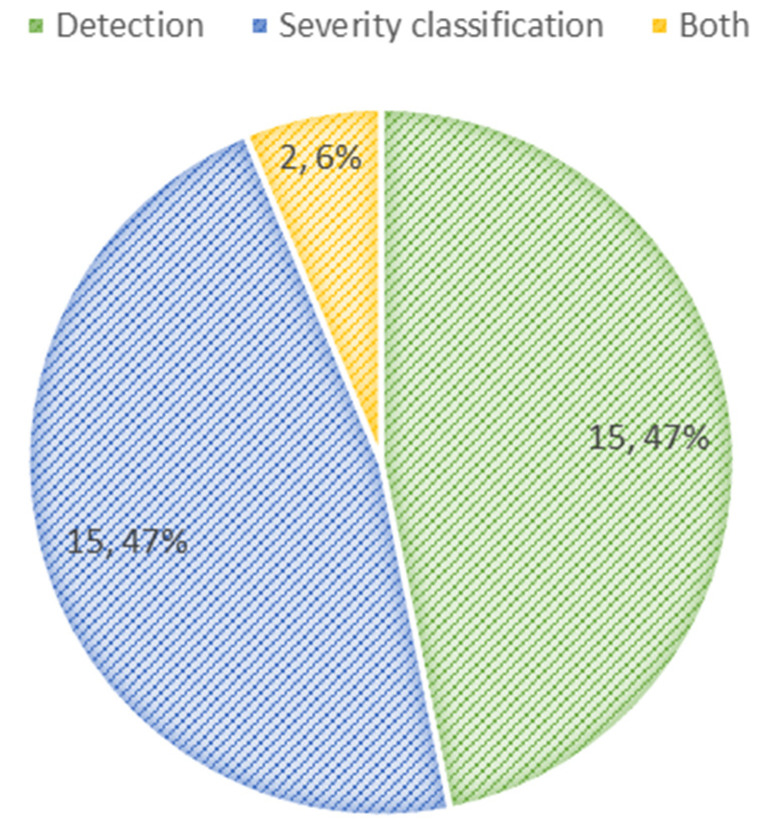
Distribution of tasks: detection, severity classification of DR, and both.

Of the 32 identified articles, 17 studies incorporated CNN-based models, two studies utilized artificial neural network (ANN)-based models, two deployed ViT-based models, and nine incorporated traditional ML algorithms into their model implementations. Also, two studies used hybrid models. A total of 15 studies aimed at detecting DR, 15 studies focused on classifying DR severity, and two studies considered both detecting and classifying DR (Figure 3).

### 3.5. Detection of DR

Fifteen studies focused on the detection of DR, while six of them used ML algorithms. Bidwai et al. reported the highest AUC of 100%, sensitivity of 84%, and accuracy of 71% using the DL technique and K-Nearest Neighbors (KNN) model [19]. Yao et al., implementing the ML technique and classification tree model, reported the lowest AUC of 72%, sensitivity of 66%, and specificity of 76% [33]. Table 2 represents a summary of included studies (Table 2).

### 3.6. Classification of DR Severity

A total of 15 studies investigated the classification of DR severity. Only one of these studies used ML algorithms. Hua et al. utilized the TFA-Net model and reported the highest AUC of 99.4% and an accuracy of 94.8% [29]. Daho et al. applied the ResNet50 model and reported an AUC of 73% [42].

### 3.7. Detection and Classification

Two studies investigated both the detection and classification of DR. Dong et al., applying multi-branch CNN, reported accuracy, sensitivity, and specificity of 96.11%, 98.08%, and 89.43%, respectively [37]. Zhou et al. reported an accuracy, sensitivity, and specificity of 99.20%, 99.49%, and 99.57% using the ViT model [44].

### 3.8. Performance Comparison of Different Models Based on Dataset Size

**Figure 4:**
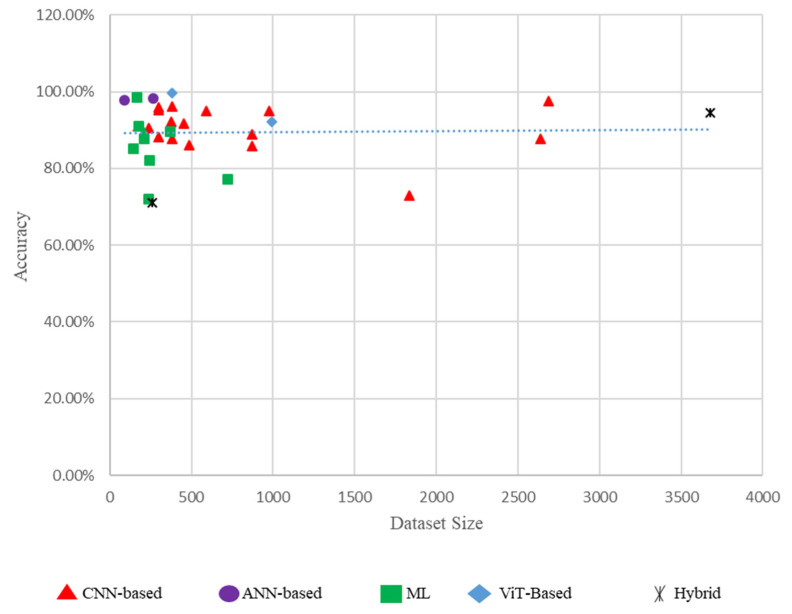
Performance comparison of different models based on dataset size.

Figure 4 illustrates the accuracy of several models in proportion to dataset size. The majority of models achieve high accuracy (surpassing 80%) despite smaller dataset sizes. This signifies that the models are adept at employing confined data. CNN-based models (red triangles) consistently exhibit enhanced accuracy across various dataset sizes. They appear to be quite helpful for screening DR. ANN-based models (purple circles) exhibit commendable accuracy but are predominantly utilized with smaller dataset sizes. This may be due to their makeup and training requirements. The traditional ML models (green squares) demonstrate a wider range of accuracies. Some models achieve high accuracy, while others demonstrate subpar performance. This diversity highlights the limitations of traditional ML methods compared to DL approaches. ViTs (blue diamonds), albeit less common, exhibit competitive performance, suggesting their potential for detecting DR.

This figure is crucial as it provides a visual comparison of the performance of several models with varied data sizes, highlighting the strengths and weaknesses of each methodology. It underscores the effectiveness of sophisticated DL models, such as CNNs and ViTs, in achieving high accuracy for DR screening (Figure 4).

### 3.9. False Positive and False Negative Rates

To enhance the validity of our findings, it is imperative to analyze the reported false positive and false negative rates in the research. The rates of false positives and false negatives show significant variability among several AI-driven models. Abdelsalam et al. [24] achieved a remarkable false negative rate of 0% and a false positive rate of 2.7%, while Yao et al. [33] reported a false negative rate of 34% and a false positive rate of 24% [44]. By employing a ViTs model, Zhou et al. [46] achieved a very low false positive rate of 0.51% and a false negative rate of 0.51%. These variations highlight the importance of thoroughly evaluating diagnostic performance, emphasizing both accuracy and the therapeutic implications of false positive and false negative rates. Accurate classification with reduced false positive and false negative rates is crucial to avoid unnecessary interventions and provide prompt treatment for affected individuals, hence improving patient outcomes in DR screening.

## 4. Discussion

The integration of AI, and especially DL, with OCTA, represents a paradigm shift in DR screening. This systematic review, encompassing 32 studies, rigorously evaluates this transformative approach, revealing the consistent superiority of DL algorithms over traditional methods for OCTA-based DR detection and classification. Beyond simply confirming the clinical utility of AI-OCTA, our discussion delves into the specific architectural innovations within DL that drive this enhanced performance, the technical nuances of OCTA imaging that are critical for AI success, and the multifaceted challenges that remain for widespread and equitable clinical implementation.

The performance advantage of DL architectures over traditional ML methods in analyzing OCTA for DR screening is evident across numerous studies. For example, studies employing CNNs, such as VGG16 [12], demonstrated notable accuracy (90.84%) and specificity (95.83%), while multilayer ANNs achieved even higher accuracy (97.78%) [21]. Although earlier ML approaches, such as support vector machines (SVMs) [24], can attain good performance (e.g., 98.5% accuracy), they often rely on handcrafted features and may not consistently match the performance levels of advanced DL models, particularly in capturing complex spatial dependencies in retinal capillaries. Advanced DL models, including Dense CNNs like DcardNet-36 [27] and ViTs [44], have shown marked performance improvements, frequently surpassing 95% accuracy, with some achieving accuracies exceeding 99% [44].

The evolution within DL architectures is noteworthy. The field has progressed from basic CNNs to more sophisticated designs incorporating multi-branch CNNs [34] and ensemble methodologies [19]. Hybrid architectures, which combine CNNs with other ML techniques such as random forests or gradient boosting machines [45], represent another promising direction. These hybrid models strategically leverage the feature extraction capabilities of CNNs with the strengths of algorithms better suited for handling tabular data or enhancing interpretability. However, claims about improved interpretability through attention maps in ViTs [44] require further validation, as the table data does not explicitly address model explainability [50].

The selection of appropriate performance metrics is critical for evaluating the clinical utility of AI-OCTA systems. While accuracy is commonly reported, sensitivity, specificity, and AUC offer a more comprehensive understanding of diagnostic performance. The variability in reported sensitivity and specificity across studies—exemplified by Li et al. [31] (sensitivity: 51.8%) and Abdelsalam et al. [24] (sensitivity: 100%)—underscores the importance of considering a balanced set of metrics. Advanced models may exhibit graded performance across DR severity levels, potentially demonstrating higher sensitivity for severe stages like proliferative DR (PDR) while facing challenges in detecting subtle early-stage non-proliferative DR (NPDR).

The technological specifications of OCTA modalities can significantly influence algorithmic outcomes. Studies utilizing swept-source OCTA (SS-OCTA) systems, such as Aslam et al. [22], suggest improved diagnostic consistency compared to spectral-domain OCTA (SD-OCTA). This is likely due to SS-OCTA’s technical advantages, including enhanced tissue penetration and reduced susceptibility to motion artifacts, which may improve visualization of the deep capillary plexus (DCP), a critical anatomical region for early DR detection [51]. Furthermore, the synergistic benefits of multimodal imaging are consistently observed across many studies. Combining OCTA with modalities such as fundus photography [29], structural OCT [19], or ultra-widefield fluorescein angiography (UWF-FA) [26] is frequently employed and associated with high diagnostic performance. Integrating OCTA data with other forms of patient data, such as clinical or demographic information [21,31], also demonstrates the potential for enhancing comprehensive DR assessment. Future research should explore optimal multimodal combinations and advanced data fusion techniques to maximize diagnostic accuracy and clinical utility.

The size and diversity of training datasets significantly influence the performance and generalizability of AI-OCTA models. Larger datasets, such as the ROAD dataset [43,47] with 2640 OCTA-DR images, enable models to learn more robust and generalizable features, as evidenced by their moderate performance metrics (accuracy: 87.5%). In contrast, smaller datasets, such as those used by Le et al. [12] (*n* = 177) and El Damrawi et al. [21] (*n* = 90), often achieve high accuracy (90.84% and 97.78%, respectively) but may overfit to specific populations or imaging protocols, limiting their applicability to broader clinical settings. For example, the Zhou et al. [44] ViT model was trained on a relatively small internal dataset (*n* = 385) and achieved exceptional accuracy (99.55%), but its generalizability remains uncertain without validation on larger, more diverse datasets. These findings underscore the importance of leveraging large, multicenter datasets to improve model robustness and reduce overfitting. Future research should prioritize the development of standardized, publicly available datasets with sufficient size and diversity to support the training of generalizable AI-OCTA models [52].

While AI models demonstrate remarkable accuracy in detecting and classifying DR, there are specific situations in which they may fail. These issues often arise from limitations in the training data, such as insufficient representation of diverse retinal conditions and inconsistencies in image capture quality. Yao et al. [33] introduced a classification tree model demonstrating an AUC of 72%, sensitivity of 66%, and specificity of 76%, encountering difficulties in accurately classifying early-stage DR. Li et al. [31] reported an accuracy of 88.10% using a ResNet50 CNN model; however, the sensitivity was notably low at 51.80%, indicating difficulties in reliably identifying actual positive instances of DR. Ryu et al. [36] employed a ResNet101 CNN model, achieving an accuracy of 72.80%, sensitivity of 67.50%, and specificity of 94.40%, highlighting challenges in detecting DR cases, particularly in the first phases. Bidwai et al. [19] utilized a hybrid model that combines DenseNet201 CNN with a neural network classifier, achieving an accuracy of 71% and a sensitivity of 84% while emphasizing potential issues around false positives. The inconsistency in OCTA devices and imaging protocols across different clinical settings can influence model performance, highlighting the need for standardized imaging practices and comprehensive datasets that encompass a variety of retinal pathologies and imaging conditions to improve the robustness and generalizability of AI models.

A key challenge identified in this review is the incompatibility of datasets, mainly due to discrepancies in OCTA devices and imaging methodologies. Multiple studies, such as those conducted by Ryu et al. [26] and Nagasawa et al. [28], highlight variations in diagnostic performance due to alterations in device settings. For example, studies utilizing SS-OCTA systems, such as the one by Aslam et al. [22], have shown improved consistency and deeper penetration compared to SD-OCTA systems. These inconsistencies may lead to fluctuations in image quality, hence affecting AI model performance, as seen by the variability in sensitivity and specificity over multiple studies. Addressing these inconsistencies requires the standardization of imaging protocols and the unification of dataset-gathering methods to ensure the generalizability and reliability of AI-based DR screening models. The replication of study findings and accessibility of datasets are critical for advancing AI-OCTA research. Publicly available datasets, such as ROAD [43,49] and EviRed [38,42], provide valuable resources for benchmarking and validating AI models across diverse populations. However, many studies in this review rely on internal datasets, such as those used by Zang et al. [27] (*n* = 303) and Ryu et al. [26] (*n* = 240), which limit the reproducibility of their findings. For instance, models trained on internal datasets, like Carrera-Escalé et al. [40] (AUC: 77%), may not generalize to other settings due to variations in imaging protocols and patient demographics. To address these challenges, researchers should prioritize open-access datasets and transparent reporting of data collection and annotation protocols. Additionally, federated learning approaches, which enable model training across multiple institutions without sharing raw data, offer a promising solution to improve dataset accessibility while maintaining patient privacy. By fostering collaboration and data sharing, the field can accelerate the development of robust, generalizable AI-OCTA systems for DR screening [53].

Achieving robust generalizability across diverse populations is a critical impediment to the widespread clinical translation of AI-OCTA systems. Current models often exhibit performance variability when applied to datasets dissimilar from their training data, raising concerns about their universal applicability. The predominance of single-center datasets in the current literature further exacerbates this issue, potentially introducing selection biases and limiting the external validity of reported performance metrics. For instance, internal datasets, as used in studies by Le et al. [12] and El Damrawi et al. [21], while achieving high accuracy within their specific context, may not reliably translate to broader clinical settings. Conversely, the performance of models trained on larger, more diverse, multicenter repositories needs to be systematically evaluated to ensure consistent and equitable performance across varied patient demographics and clinical environments. Addressing class imbalance within datasets, as encountered in studies with unequal ratios of DR to control cases, also requires careful consideration, potentially necessitating techniques like synthetic oversampling to mitigate bias and improve the detection of subtle early-stage DR [52].

Translating AI-OCTA systems from controlled research settings into routine clinical practice introduces significant translational challenges. Workflow integration represents a primary practical consideration. While AI-driven analysis promises to expedite image interpretation, pilot implementations require robust computational infrastructure to maintain acceptable inference latency, particularly in high-throughput clinical environments. For example, models like Zang et al. [39] 3D CNN (accuracy: 95%) and Zhou et al. [44] ViT (accuracy: 99.55%) demonstrate high performance but require significant computational resources, which may not be readily available in resource-limited settings. Furthermore, adapting AI-OCTA for telemedicine applications while offering the potential to expand DR screening access to underserved populations faces challenges related to image acquisition quality in real-world settings. As highlighted by the need for quality assessment modules, ensuring consistent image quality from diverse OCTA devices (e.g., Optovue, Topcon, Zeiss) and in less controlled environments is crucial for robust AI performance.

Integrating AI-OCTA models with clinician expertise can significantly enhance diagnostic performance. AI aids clinicians by flagging areas of interest, providing decision support, and helping interpret images, especially in complex cases. Combining OCTA with other imaging modalities offers a comprehensive view of the patient’s condition and reduces diagnostic variability due to image quality and clinician experience. The AI-clinician collaboration reduces cognitive load and accelerates accurate decision-making, driving efficient, high-quality DR management.

Beyond technical and workflow challenges, ethical and legal considerations are paramount. The lack of widely recognized regulatory approval pathways for AI-OCTA in DR screening reflects unresolved issues related to validation standards, clinical acceptance criteria, and ethical oversight. For instance, models trained on internal datasets, such as Carrera-Escalé et al. [40] (AUC: 77%), may not meet the rigorous validation requirements for regulatory approval, raising concerns about bias, generalizability, and patient safety. Ethical concerns also arise regarding data privacy, informed consent, and the potential for algorithmic bias, particularly when deploying AI-OCTA systems in diverse populations. Standardization of image acquisition protocols, data annotation, and performance reporting are essential not only to facilitate comparability across studies but also to build clinician trust and ensure equitable access to AI-driven diagnostic support. Addressing these ethical and legal challenges is critical to ensuring the responsible and equitable implementation of AI-OCTA systems in clinical practice [54].

The sustainable adoption of AI-OCTA in DR screening necessitates careful consideration of cost-benefit dynamics. While the initial investment in AI infrastructure and model development can be substantial, the potential for long-term cost savings through improved screening efficiency and reduced burden on human graders is significant. For example, hybrid models like Bidwai et al. [45] (ResNet-101 + LightGBM: accuracy: 94.32%) and multimodal approaches like Hua et al. [29] (OCTA + fundus image: accuracy: 94.8%) demonstrate the ability to process large volumes of imaging data efficiently, reducing the need for manual interpretation. Furthermore, the potential to improve screening accessibility, particularly in resource-limited settings and through telemedicine-enabled AI-OCTA, offers a pathway to reduce healthcare disparities and improve population-level DR management. However, comprehensive health economic evaluations are crucial to demonstrate the cost-effectiveness of AI-OCTA compared to existing screening paradigms across diverse healthcare systems and DR prevalence scenarios. For instance, models trained on public datasets like ROAD [43,49] and EviRed [38,42] show promise but require further validation in real-world settings to assess their economic viability. Reimbursement models that incentivize the adoption of AI-driven diagnostic tools and recognize their value in improving patient outcomes are also essential to facilitate widespread clinical integration and realize the full potential of AI-OCTA in transforming DR care [55].

## 5. Conclusions

In conclusion, the integration of AI with OCTA holds immense promise for revolutionizing DR screening, offering the potential for enhanced accuracy, efficiency, and accessibility. DL models, particularly advanced architectures like ViTs [44] and hybrid approaches [45], demonstrate superior performance compared to traditional methods and manual grading. These AI-OCTA systems can significantly impact clinical practice by streamlining workflows, reducing diagnostic delays, and enabling scalable screening programs, particularly in underserved and resource-limited settings. By automating image analysis and providing quantitative metrics, AI-OCTA systems can alleviate the burden on healthcare professionals, allowing them to focus on complex cases and patient care.

However, realizing this transformative potential requires addressing key challenges. Generalizability remains a critical concern, as many AI-OCTA models are trained on single-center or homogeneous datasets, limiting their applicability to diverse populations and clinical environments. Ethical challenges, including data privacy, algorithmic bias, and informed consent, must also be carefully navigated to ensure equitable and responsible implementation. Regulatory and standardization hurdles further complicate the translation of AI-OCTA systems into routine clinical practice, underscoring the need for robust validation frameworks and transparent reporting.

In conclusion, AI-OCTA models can enhance diagnostic efficiency and accuracy in DR management. By supporting clinicians with faster analysis and consistent results, AI helps streamline workflows and offers a more personalized approach to patient care. This collaboration leads to improved decision-making and better patient outcomes, ultimately reducing the burden of DR-related vision loss.

## Figures and Tables

**Table 1:** Search strategies for each database.

Database	Search String	Results
Pubmed	(((((((((“Neural Networks, Computer”) OR “Deep Learning”) OR “Artificial Intelligence”) OR (“Machine Learning” OR “Unsupervised Machine Learning” OR “Supervised Machine Learning”)) OR (neural network)) OR (convolutional neural network)) OR (“Computers”)) OR (predictive markers[Title/Abstract])) AND (((((“Retinal Diseases” OR “Hypertensive Retinopathy” OR “Diabetic Retinopathy”) OR (“Diabetes Mellitus” OR “Diabetes, Gestational” OR “Diabetes Mellitus, Type 1” OR “Diabetes Mellitus, Type 2”)) OR (“Eye Diseases”)) OR (“Eye”)) OR (“Neovascularization, Pathologic”))) AND ((((((((((OCTA) OR (oct angiography)) OR (oct-angiography)) OR (Optical coherence tomography angiography)) OR ((“Angiography/classification” OR “Angiography/methods”))) OR (“Tomography, Optical Coherence/methods”)) OR (En Face OCT)) OR (Swept-Source OCT)) OR (OCT angiogram[Title/Abstract])) OR (angiographic OCT))	824
Scopus	(TITLE-ABS-KEY(“Neural Networks, Computer” OR “Deep Learning” OR “Artificial Intelligence” OR “Machine Learning” OR “Unsupervised Machine Learning” OR “Supervised Machine Learning” OR “neural network” OR “convolutional neural network” OR “Computers” OR “predictive markers”)) AND (TITLE-ABS-KEY(“Retinal Diseases” OR “Hypertensive Retinopathy” OR “Diabetic Retinopathy” OR “Diabetes Mellitus” OR “Gestational Diabetes” OR “Type 1 Diabetes Mellitus” OR “Type 2 Diabetes Mellitus” OR “Eye Diseases” OR “Eye” OR “Pathologic Neovascularization”)) AND (TITLE-ABS-KEY(OCTA OR “oct angiography” OR “oct-angiography” OR “Optical Coherence Tomography Angiography” OR “Angiography classification” OR “Angiography methods” OR “Optical Coherence Tomography methods” OR “En Face OCT” OR “Swept-Source OCT” OR “OCT angiogram” OR “angiographic OCT”))	848
Web Of Science	TS = ((“artificial intelligence” OR “machine learning” OR “deep learning” OR “neural network*” OR “convolutional neural network*”) AND (“diabetes” OR “type 1 diabetes” OR “type 2 diabetes” OR “diabetic complication*” OR “diabetic management”) AND (“optical coherence tomography angiography” OR “optical coherence tomography” OR “angiography” OR “retinal imaging”))	236
Embase	(‘artificial intelligence’/exp OR ‘machine learning’/exp OR ‘deep learning’/exp OR ‘neural networks’/exp OR ‘convolutional neural network’/exp) AND (‘diabetes’/exp OR ‘type 1 diabetes’/exp OR ‘type 2 diabetes’/exp OR ‘diabetic complications’/exp OR ‘diabetic management’) AND (‘optical coherence tomography angiography’/exp OR ‘angiography’/exp OR ‘retinal imaging’/exp)	1075

**Table 2:** Extracted Data with Best Results per Article.

Author	Year	Training Database	Type Image	Imaging Modality	Number of Images	Outcome	Algorithm/Model Used	Performance Metrics
**Le et al.** [12]	2020	Internal	OCTA	AngioVue, Optovue, Fremont, CA, USA	177	Healthy, No DR, DR	CNN-Based: VGG16 CNN	ACC: 90.84 SPE: 95.83
**El Damrawi et al.** [21]	2020	Internal	OCT-A	OCTA (Triton Topcon SS-OCTA, 1050 nm wavelength, Topcon Corporation, Tokyo, Japan))	90	Normal, No DR, NPDR (mild to moderate)	ANN-Based: Multistage ANN (MANN)	ACC: 97.78% SEN: 96.67%
**Aslam et al.** [22]	2020	Internal	SS-OCTA	OCTA (Topcon Swept-Source Triton DRI-OCT, 100,000 A-scan rate, Topcon Corporation, Tokyo, Japan))	182	No Diabetes, No DR, DR	Traditional ML: Random Forest	AUC: 91%
**Heisler et al.** [23]	2020	Internal	Multi-modal (OCTA + Structural OCT)	OCTA (Zeiss Plex Elite, 3 × 3 mm protocol, Carl Zeiss Meditec AG, Jena, Germany)	380	Referable, non-referable DR	CNN-Based: Ensemble (4× VGG19 CNNs)—Majority Soft Voting	ACC: 92.00% AUC: 92% SEN: 90.40% SPE: 93.30%
**Abdelsalam et al.** [24]	2021	Internal	OCTA	Not specified	170	Early NPDR vs. Healthy Eyes	Traditional ML: SVM	ACC: 98.50% SEN: 100% SPE: 97.30%
**Liu et al.** [25]	2021	Internal	OCTA	Optovue OCTA, Optovue Inc., Fremont, CA, USA	246	DR, Healthy Eyes	Traditional ML: Wavelet Features + LR-EN, LR, SVM, XGBoost	ACC: 82% AUC: 84% SEN: 84% SPE: 80%
**Ryu et al.** [26]	2021	Internal	Multimodal (OCTA and UWF-FA)	OCTA: Optovue RTVue XR AVANTI, Optovue Inc., Fremont, CA, USA; UWF FA: Optos California, Optos plc, Dunfermline, UK	240	Healthy eyes, mild NPDR, moderate NPDR, severe NPDR, PDR	CNN-Based: ResNet101 CNN	ACC: 90.40% AUC: 94.6% SEN: 93.10% SPE: 85.00%
**Zang et al.** [27]	2021	Internal	Multi-modal (OCT and OCTA)	RTVue-XR Avanti SD-OCT, Optovue Inc., Fremont, CA, USA	303	Three classification levels based on the International Clinical DR scale: -Level 1: 2 classes (non-referable DR, referable DR) -Level 2: 3 classes (no DR, NPDR, PDR) -Level 3: 4 classes (no DR, mild/moderate NPDR, severe NPDR, PDR)	CNN-Based: DcardNet-36 (Dense CNN)	ACC: 95.70% SEN: 91.00% SPE: 98.00%
**Nagasawa et al.** [28]	2021	Internal	Multimodal (UWF-FA and OCTA)	UWF Fundus Ophthalmoscopy: Optos 200Tx^®^, Nikon Corporation, Tokyo, Japan; OCTA: OCT Triton Plus^®^, Topcon Corporation, Tokyo, Japan	491	No DR, Mild and moderate NPDR, Severe NPDR, PDR	CNN-Based: Deep CNN (VGG16)	ACC: 86% AUC: 92.8% SEN: 74.50% SPE: 97.00%
**Hua et al.** [29]	2021	Internal and Messidor dataset	Multimodal (Fundus images and SS-OCTA)	Fundus Imaging: Optos 200Tx^®^, Nikon Corporation, Tokyo, Japan; SS-OCTA: OCT Triton Plus^®^, Topcon Corporation, Tokyo, Japan	594	No DR, Mild and moderate NPDR, Severe NPDR, PDR	CNN-Based: TFA-Net (ResNet-18 Backbone)	ACC: 94.80% AUC: 99.4%
**Guo et al.** [30]	2021	Internal and Messidor dataset	Fundus Imaging: Optos 200Tx^®^ by Nikon; SS-OCTA: OCT Triton Plus^®^ by Topcon	OCTA (AngioVue, Optovue, Fremont, CA, USA))	978	No DR, Mild and moderate NPDR, Severe NPDR, PDR	CNN-Based: U-Net-like CNN (ResNet Residual Modules)	ACC: 94.8% AUC: 99.4%
**Li et al.** [31]	2022	Internal	Multimodal (Fundus images and SS-OCTA)	Fundus Imaging: Optos 200Tx^®^, Nikon Corporation, Tokyo, Japan; SS-OCTA: VG200D, SVision Imaging, Ltd., Luoyang, China	386	Normal, NPDR, PDR	CNN-Based: DenseNet121, EfficientNet-b3	AUC: 87.61%
**Li et al.** [32]	2022	OCTA-500 dataset	OCTA	SS-OCTA system: VG200D, SVision Imaging, Ltd., Luoyang, China	301	Normal, NPDR, PDR	CNN-Based: ResNet50 CNN	ACC: 88.10% AUC: 92% SEN: 51.80% SPE: 96.30%
**Yao et al.** [33]	2022	Internal	OCTA	SS-OCTA System: VG200D, SVision Imaging, Ltd., Luoyang, China	241	1. Diabetes, Healthy 2. Referable DR, Non-referable DR 3. Severe DR, Non severe DR	Traditional ML: Classification Tree	AUC: 72% SEN: 66% SPE: 76%
**Zang et al.** [34]	2022	Internal	Multi-modal (OCT and OCTA)	SD-OCT system RTVue-XR Avanti, Optovue Inc., Fremont, CA, USA	456	non-referable DR, referable DR, vision-threatening DR, NPDR, referable DR but non-vision threatening DR, vision-threatening DR	CNN-Based: 3D CNN	ACC: 91.52% AUC: 96% SEN: 90.77% SPE: 92.50%
**Hou et al.** [35]	2022	DRAC dataset	Multimodal (OCTA, Color Fundus Photography)	SD-OCT system RTVue-XR Avanti, Optovue Inc., Fremont, CA, USA	997	Non-referable DR, Referable DR, Vision threatening DR	ViT-Based: Pre-trained on EyePACS & DDR	ACC: N/A AUC: 92% SEN: N/A SPE: 86%
**Khalili Pour et al.** [20]	2022	Internal	OCTA	OCTA (RTVue XR 100 Avanti, Optovue Inc., Fremont, CA, USA)	148	NPDR, PDR	Traditional ML: SVM Optimized by Genetic Algorithm	ACC: 85% AUC: N/A SEN: N/A SPE: N/A
**Ryu et al.** [36]	2022	Internal	Multimodal (OCTA and demographic data (age and gender))	Optovue RTVue XR Avanti, Optovue Inc., Fremont, CA, USA	1835	Normal, No DR, Mild NPDR, Moderate NPDR, Severe NPDR, PDR	CNN-Based: ResNet101 CNN	ACC: 72.80% SEN: 67.50% SPE: 94.40%
**Dong et al.** [37]	2022	Internal	OCTA, UWF-FA	SS-OCT system RTVue-XR Avanti, Optovue Inc., Fremont, CA, USA	385	No DR, Mild NPDR, Moderate to Severe NPDR, PDR	CNN-Based: Multi-Branch CNN (Inception-V3 & VGG16)	ACC: 96.11% AUC: 94.6% SEN: 93.10% SPE: 85.00%
**Li et al.** [38]	2023	EviRed dataset	OCTA	OCTA (PLEX^®^Elite 9000, Carl Zeiss Meditec Inc., Dublin, CA, USA)	875	No DR, Mild NPDR, Moderate to Severe NPDR, PDR	CNN-Based: 3D CNN Ensemble (ResNet, DenseNet, EfficientNet) + Hierarchical Fusion	ACC: 88.68% AUC: 88.68%
**Zang et al.** [39]	2023	Internal	Multi-modal (Structural OCT and OCTA)	OCTA (Avanti RTVue-XR, Optovue Inc., Fremont, CA, USA)	302	Normal, DR, Age-related macular degeneration, Glaucoma	CNN-Based: Custom 16-Layer 3D CNN	ACC: 95%
**Carrera-Escalé et al.** [40]	2023	Internal	Multimodal (Fundus Retinography, OCT, and OCTA)	Topcon DRI-Triton, Topcon Corporation, Tokyo, Japan; Cirrus 5000, Carl Zeiss Meditec AG, Jena, Germany; Angioplex Zeiss, Carl Zeiss Meditec AG, Jena, Germany	726	Normal, DM, DR, Referable DR	Traditional ML: LR, LDA, SVC, RF	AUC: 77%
**El Damrawi et al.** [41]	2023	Internal	SS-OCTA	SS-OCTA (Triton TopCon SS-OCTA, Topcon Corporation, Tokyo, Japan)	270	Normal, No DR, NPDR, PDR	ANN-Based: Multistage ANN	ACC: 98.10% SEN: 96.67–100% SPE: 96–100%
**Daho et al.** [42]	2023	EviRed dataset	Multi-modal (UWF Color Fundus Photography and OCTA)	OCTA (Clarus 500, Carl Zeiss Meditec, Dublin, CA, USA; PLEX Elite 9000, Carl Zeiss Meditec, Dublin, CA, USA)	875	Normal, Mild NPDR, Moderate NPDR, Severe NPDR, PDR, Pan-Retinal Photocoagulation	CNN-Based: Multimodal Deep Fusion (SE-ResNet50 & SE-3D-ResNet50)	ACC: 85.66% AUC: 80.37% SEN: 79.22% SPE: 88.20%
**Ma et al.** [43]	2023	ROAD dataset	OCTA	OCTA (Widefield SS-OCT System, VG200D; SVision Imaging, Ltd., Luoyang, China)	2640 (OCTA-DR data)	Normal, Mild NPDR, PDR	CNN-Based: PACNet (Projective Map Attention CNN)	ACC: 87.5% AUC: N/A SEN: N/A SPE: N/A
**Zhou et al.** [44]	2023	Internal	WF-OCTA	WF-OCTA (SS-OCT System, VG200D; SVision Imaging, Ltd., Luoyang, China)	385	No DR, Mild NPDR, Moderate to Severe NPDR, PDR	ViT-Based	ACC: 99.55% SEN: 99.49% SPE: 99.57%
**Bidwai et al.** [45]	2024	Internal	Multimodal (UWF Color Fundus Photography and OCTA)	UWF Color Fundus Photography and OCTA (Eidon Machine, iCare Finland Oy, Vantaa, Finland; Optovue Avanti, Optovue, Inc., Fremont, CA, USA)	3680(UWF Color Fundus Photography) 3288(OCTA)	Normal DR (various severity levels)	Hybrid: ResNet-101 CNN + DkSO-Optimized LightGBM	ACC: 94.32% SEN: 94.94% SPE: 94.78%
**Li et al.** [46]	2024	Internal	Multimodal (OCTA and Clinical data)	OCTA (Optovue, Inc., Fremont, CA, USA)	372	No DR, Mild NPDR, Moderate NPDR, Severe NPDR, Referable DR, Vision threatening DR	Traditional ML: Random forest	ACC: 89.40% AUC: 96.65%
**Bidwai et al.** [19]	2024	Internal	Multi-modal (UWF Color Fundus and OCTA)	OCTA (Optovue Avanti Edition, Optovue, Inc., Fremont, CA, USA))	262	Normal DR (various severity levels)	Hybrid: Pre-trained DenseNet201 CNN + Neural Network Classifier	ACC: 71% AUC: 100% SEN: 84% SPE: N/A
**Abtahi et al.** [47]	2024	Internal	OCTA	OCTA (AngioVue SD-OCT, Optovue, Fremont, CA, USA)	212	Normal, No DR, Mild NPDR, Moderate NPDR, Severe NPDR	Traditional ML: SVM + SFS + CLV	ACC (Binary): 87.63% SEN (Binary): 89.70%
**Abtahi et al.** [48]	2024	Internal	OCTA	OCTA (AngioVue SD-OCT, Optovue, Inc., Fremont, CA, USA))	212	Normal, No DR, Mild NPDR, Moderate NPDR, Severe NPDR	Traditional ML: SVM + SFS + AVA-Net	ACC: 89.26% (Binary) AUC: 87.23%
**Ma et al.** [49]	2024	ROAD dataset	OCTA	OCTA (SS-OCT System, manufacturer details not specified))	2693 (OCTA-DR data)	Normal, Mild NPDR, PDR	CNN-Based: CSANet (Channel & Spatial Attention CNN)	ACC: 97.41% AUC: N/A SEN: N/A SPE: N/A

**ACC;** Accuracy, **SPE;** Specificity, **SEN;** Sensitivity, **AUC;** Area under the curve, **PDR;** Proliferative diabetic retinopathy, **NPDR;** Non proliferative diabetic retinopathy.

## Data Availability

Data are available from the corresponding author upon reasonable request.

## Supplementary Materials

The following supporting information can be downloaded at: https://www.mdpi.com/article/10.3390/diagnostics15060737/s1, Supplement S1. Quality assessment of included studies. Supplement S2. Detailed overview of public databases used in included studies [56].

## Abbreviations

**AI**	Artificial Intelligence
**ANN**	Artificial Neural Network
**AUC**	Area Under the Curve
**CNN**	Convolutional Neural Network
**DCNN**	Deep Convolutional Neural Network
**DenseNet121**	Dense Convolutional Network 121
**DL**	Deep Learning
**DR**	Diabetic Retinopathy
**DRAC**	Diabetic Retinopathy and Cataract dataset
**FA**	Fluorescein Angiography
**KNN**	K-Nearest Neighbors
**LR**	Logistic Regression
**LightGBM**	Light Gradient Boosting Machine
**LDA**	Linear Discriminant Analysis
**ML**	Machine Learning
**NPDR**	Non-Proliferative Diabetic Retinopathy
**OCTA**	Optical Coherence Tomography Angiography
**PDR**	Proliferative Diabetic Retinopathy
**ResNet**	Residual Network
**SFS**	Sequential Feature Selection
**SVM**	Support Vector Machine
**SS-OCTA**	Swept-Source Optical Coherence Tomography Angiography
**TFA-Net**	Transformer-based Feature Attention Network
**UWF-FA**	Ultra-Widefield Fluorescein Angiography
**ViT**	Vision Transformer
**XAI**	Explainable Artificial Intelligence
**XGBoost**	Extreme Gradient Boosting
**VGG16**	Visual Geometry Group 16
**SE-ResNet50**	Squeeze-and-Excitation ResNet50
