# Supplementary material for: Advancing Diabetic Retinopathy Screening: A Systematic Review of Artificial Intelligence and Optical Coherence Tomography Angiography Innovations
Source: Diagnostics (Basel). 2025 Mar 15;15(6):737. doi: 10.3390/diagnostics15060737 (PMC11941001; doi:10.3390/diagnostics15060737)
Supplement: Supplementary file 1 [file diagnostics-15-00737-s001.zip › Supplement S1.pdf]

Supplement S1. Quality assessment of included studies

Study<br>First author (year)	Selection					Comparability			Exposure				Total
	Case definition adequacy	Representative of cases	Selection of controls	Definition of controls	Subtotal	Age	Other factors	Subtotal	Ascertainment of exposure	Same ascertainment	Non-response rate	Subtotal	
1. Aslam (2020)	*	*	*	*	4	*	*	2	*	*	*	3	9
2. El Damrawi (2020)	*	*	*	*	4	*	*	2	*	*		2	8
3. Heisler (2020)	*	*			2			0	*	*	*	3	5
4. Le (2020)	*	*	*	*	4	*	*	2	*	*	*	3	9
5. Abdelsalam(2021)	*	*		*	3	*		1	*	*	*	3	7
6. Guo (2021)	*		*	*	3			0	*	*	*	3	6
7. Hua (2021)	*		*		2			0	*	*	*	3	5
8. Liu (2021)	*	*	*	*	4			0	*	*	*	3	7
9. Nagasawa (2021)	*	*			2	*	*	2	*	*		2	6
10. Ryu (2021)	*	*	*	*	4			0	*	*	*	3	7
11. Zang (2021)	*	*	*	*	4			0	*	*		2	6
12. Dong (2022)	*	*	*	*	4			0	*	*	*	3	7
13. Hou (2022)	*		*		2			0	*	*	*	3	5
14. Khalili Pour (2022)	*	*			2	*	*	2	*	*	*	3	7
15. Yihao Li (2022)	*		*		2	*		1	*		*	2	5
16. Qiaoyu Li (2022)	*				1		*	1	*	*	*	3	5
17. Ryu (2022)	*	*	*	*	4	*		1	*	*	*	3	8
18. Yao (2022)	*	*			2	*	*	2	*		*	2	6
19. Zang (2022)	*	*	*	*	4		*	1	*	*	*	3	8
20. Carrera (2023)	*	*	*	*	4		*	1	*	*	*	3	8
21. Daho (2023)	*	*	*		3			0	*	*	*	3	6
22. El Damrawi (2023)	*	*	*	*	4	*	*	2	*	*	*	3	9
23. Yihao Li (2023)	*	*			2			0	*	*	*	3	5
24. Ma (2023)	*		*		2			0	*	*	*	3	5

25. Zang (2023)	*	*	*	*	4			0	*	*		2	6
26. Zhou (2023)	*	*	*	*	4			0	*		*	2	6
27. Abtahi (2024) doi.org/10.1167/iovs.65.10.20	*	*	*	*	4	*	*	2	*	*	*	3	9
28. Abtahi (2024) doi.org/10.1364/BOE.521657	*	*	*	*	4	*	*	2	*	*	*	3	9
29. Bidawi (2024) doi.org/10.1016/j.mex.2024.102910	*	*	*	*	4			0	*		*	2	6
30. Bidwai (2024) doi.org/10.1016/j.inffus.2024.102526	*	*			2			0	*	*	*	3	5
31. Xiaoli Li (2024)	*	*	*		3	*	*	2	*		*	2	7
32. Ma (2024)	*		*		2			0	*	*	*	3	5

Stars are allocated to articles that fulfill the criteria of Newcastle-Ottawa scale (NOS) in each field.
