# Supplementary material for: Advancing Diabetic Retinopathy Screening: A Systematic Review of Artificial Intelligence and Optical Coherence Tomography Angiography Innovations
Source: Diagnostics (Basel). 2025 Mar 15;15(6):737. doi: 10.3390/diagnostics15060737 (PMC11941001; doi:10.3390/diagnostics15060737)
Supplement: Supplementary file 1 [file diagnostics-15-00737-s001.zip › Supplement S2.pdf]

Supplement S2. Detailed overview of public databases used in included studies

Dataset	Number of images	Acquired from	Ground truth labelling	DR definition methods
**Messidor**	1200	3 ophthalmologic departments	two specialists	• 0: (Normal): $(\mu A = 0)$ AND $(H = 0)$<br>• 1: $(0 < \mu A <= 5)$ AND $(H = 0)$<br>• 2: $((5 < \mu A < 15)$ OR $(0 < H < 5))$ AND $(NV = 0)$<br>• 3: $(\mu A >= 15)$ OR $(H >=5)$ OR $(NV = 1)$
**OCTA-500**	500	Jiangsu Province Hospital from March 2018 to July 2020	Ophthalmologists	NM
**DRAC**	1,103	NM	two ophthalmologists	DR severity scale[1]
**EviRed**	5000	18 diabetology departments and 14 ophthalmology departments	two graders in the EviRed virtual reading centre	presence of proliferative DR and/or significant macular oedema involving the centre of the macula, or need for laser photocoagulation, vitrectomy or intravitreal injection depending on updated indication of these treatments
**ROAD**	1200 no DR<br>1440 DR<br>1440 ground truth of segmentation for DR	based on DRAC in 2022	professional ophthalmologists	No DR, NPDR, PDR

1. Organization, W.H., *Prevention of blindness from diabetes mellitus: report of a WHO consultation in Geneva, Switzerland, 9-11 November 2005*. 2006: World Health Organization.
